# Lipidomic analysis of human plasma reveals ether-linked lipids that are elevated in morbidly obese humans compared to lean

**DOI:** 10.1186/1758-5996-5-24

**Published:** 2013-05-14

**Authors:** Elise L Donovan, Stefan M Pettine, Matthew S Hickey, Karyn L Hamilton, Benjamin F Miller

**Affiliations:** 1 Department of Health and Exercise Science, Colorado State University, Fort Collins, CO 8052, USA; 2 Bariatric Center of the Rockies, Fort Collins, CO, USA; 3 The Liggins Institute, University of Auckland, 85 Park Rd Grafton, Auckland, NZ 1142, New Zealand

**Keywords:** Lipidomics, Obesity, Dyslipidemia, Endothelial cells, Oxidized phospholipids

## Abstract

**Background:**

Lipidomic analysis was performed to explore differences in lipid profiles between plasma from lean and obese subjects, followed by *in vitro* methods to examine a role for the identified lipids in endothelial cell pathophysiology.

**Methods:**

Plasma was collected from 15 morbidly obese and 13 control subjects. Lipids were extracted from plasma and analyzed using LC/MS, and MS/MS to characterize lipid profiles and identify lipids that are elevated in obese subjects compared to lean.

**Results:**

Orthogonal partial least squares-discriminant analysis (OPLS-DA) modelling showed that lipid profiles were significantly different in obese subjects compared to lean. Analysis of lipids that were driving group separation in the OPLS-DA model and that were significantly elevated in the obese group led to identification of a group of ether-linked phosphatidylcholine (PC) and phosphatidylethanolamine (PE) lipids of interest. Treatment of human coronary artery endothelial cells with the ether-linked phosphatidylethanolamine induced expression of cell adhesion molecules, a hallmark of endothelial cell activation. However, oxidized phosphatidylcholine products that can induce endothelial cell activation *in vitro*, were not significantly different between groups *in vivo*.

**Conclusion:**

These data suggest a role for ether-linked lipids in obesity associated dyslipidemia and vascular disease.

## Background

Dyslipidemia and oxidative stress are characteristic of obesity and diabetes, and appear to play a major role in diabetes, cardiovascular disease, and cancer [1,2]. Obesity is associated with increased circulating lipids, and increased oxidized LDL that leads to inflammation and oxidative stress in vascular endothelial cells and contributes to the pathophysiology of the metabolic syndrome and atherosclerosis [3,4]. Ectopic fat deposition in morbidly obese individuals also contributes to the pathogenesis of vascular and metabolic disturbances by inhibiting insulin action and disrupting lipid metabolism in the adipose, liver, pancreas, kidney and skeletal, smooth and cardiac muscle [5]. Phosphatidylcholine (PC) species that contain polyunsaturated fatty acids, particularly arachidonate, at the sn-2 position are especially susceptible to free radical oxidation [6]. One such PC is 1-palmitoyl-2-arachidonoyl-sn-glycero-3-phosphorylcholine (PAPC), which is a common cell membrane constituent, and circulates within cholesterol particles. PAPC and several products of PAPC oxidation including 1-palmitoyl-2-glutaroyl-sn-glycero-3-phosphorylcholine (PGPC), 1-palmitoyl-2-(5,6-epoxyisoprostane E_2_)-sn-glycero-3-phosphorylcholine (PEIPC),1-palmitoyl-2-oxovaleroyl-sn-glycero-3-phosphorylcholine (POVPC), and lysophosphatidylcholine (lyso-PC) have been shown to induce cell adhesion molecule expression and inflammatory mediator secretion in endothelial cells and have been implicated in atherosclerotic progression [7-12], and oxidized phospholipids are found deposited in atherosclerotic plaques [6,13]. Because PC is the most abundant cellular phospholipid, most oxidation products detected contain the choline head group [14]. However, other lipid classes and additional oxidized phospholipids are likely involved in vascular pathology, and it is likely that interactive effects between lipids contribute to CVD/metabolic disease pathophysiology.

In addition to (oxidized) phospholipids, other lipids including but not limited to saturated and polyunsaturated fatty acids and ether-linked lipids have been examined in the relation to metabolic syndrome and cardiovascular disease. Ether-linked lipids, characterized by an ether linkage between the glycerol backbone and one or both fatty acid side chains (usually the sn-1 position) as opposed to an ester linkage, and plasmalogens, a subclass of ether-linked lipids characterized by a vinyl ether linkage at the sn-1 position with an ester linkage at the sn-2 position, have also been the subject of recent research. Plasmalogens may function as free radical scavengers, and ether-linked lipids can serve as arachidonic acid reservoirs in cell membranes [15-17]. The role of ether-linked lipids and plasmalogens in metabolic and cardiovascular disease is still not well understood as there is lack of concensus in the data as to patterns of change in ether-linked lipid distribution with different metabolic syndrome characteristics [18,19].

Lipidomics is broadly defined as the large-scale study of pathways and networks of cellular lipids in biological systems. Lipidomics can be used to examine the presence and structure of the range of lipid species within different tissues making it an attractive method for examining distribution of phospholipids in tissue, as well as for globally examining differences in lipids between subject groups. A broad examination of lipid species that are elevated and potentially contribute to metabolic and cardiovascular disease pathologies in morbidly obese humans could provide insight into the link between obesity and pathogenesis of atherosclerosis and facilitate discovery of lipid biomarkers that increase disease risk.

The purpose of this study was to: 1) use a shotgun lipidomics approach to examine global lipid distribution in plasma from lean and morbidly obese humans, to identify lipids that are elevated in the morbidly obese population that may contribute to metabolic and cardiovascular pathology, 2) examine the effects of lipids identified in our shotgun analysis as elevated in obese subjects in an *in vitro* endothelial cell model to determine whether they induce disease related phenotypic changes, and 3) use a targeted lipidomics approach to determine if there is a difference in distribution of oxidized phospholipids in plasma from lean and morbidly obese humans. We hypothesized that lipid profiles would be significantly different in plasma from morbidly obese humans compared to lean, and that lipids identified in our shotgun approach that are elevated in obese subjects compared to lean would induce inflammatory changes in endothelial cells. In addition, we hypothesized that oxidized phospholipids shown previously *in vitro* to cause endothelial cell production of inflammatory mediators and cell adhesion molecules would be greater in plasma from obese humans compared to lean.

## Materials and methods

### Ethics statement

The Institutional Review Boards of Colorado State University and Poudre Valley Hospital approved this protocol (CSU protocol #- 05-116H, PVH protocol #- 07–874). Each volunteer was informed of the potential risks and written consent was obtained prior to enrolment. The study followed the guidelines set forth by the Declaration of Helsinki.

### Study overview

A total of 15 morbidly obese gastric bypass patients, and 13 non-obese controls age 18–60 were recruited to participate through the Northern Colorado Surgical Associates (NCSA) of the Bariatric Center of the Rockies. Bypass patients were required to have a body mass index (BMI) > 40 kg/m^2^, while controls were required to have a BMI < 30 kg/m^2^. There were no other exclusion criteria allowing the subject population to be heterogeneous and, thus, differences in lipid profiles between groups are representative of the general clinical population. Subjects completed medical and exercise questionnaires prior to undergoing a venipuncture blood draw following a 12-hour fast. Blood was collected in vacutainer tubes containing EDTA, 0.5 mL were aliquoted for HbA1C analysis, and the remainder was centrifuged (1200 g, 15 min, 4°C) for collection of plasma which was stored at −80°C until lipid extractions were performed.

### Subject characteristics

Participant characteristics are shown in Table 1. We were unable to obtain body composition data because subject size exceeded the capacity of the available dual energy x-ray absorptiometry (DEXA) equipment. Height and weight were measured at the surgical center, while other demographic and health information was obtained through a health history questionnaire. None of the subjects were undergoing active weight reduction prior to surgery. Whole blood samples were sent to the University of Colorado Denver Clinical Translational Research Center for analysis of hemoglobin A1C (HbA1C) using a DCA Vantage analyzer (Siemens, Deerfield, IL).

**Table 1:** Subject characteristics

	**Control**	**Obese**
	**n = 13**	**n = 15**
	**(F = 11, M = 2)**	**(F = 10, M = 5)**
Age (yr)	42	44
Height (in)	65 ± 3	67 ± 3
Weight (kg)	71 ± 14	142 ± 33 *
BMI (kg/m^2^)	25.76 ± 4.39	49.87 ± 11.27 *
BMI min	20	41
BMI max	31	87
HbA1C (% of total)	5.15 ± 0.10	6.41 ± 0.35 *
Current smokers	2	1
Former smokers	4	11
Statins	2	1
Anti-Hypertensives	2	6
Hypoglycemics	0	4
Anti-anxiety and anti-depressants	4	5

* = p < 0.01 compared to control. Data are presented as Mean ± SE.

### Lipid extraction

Lipid extractions were performed using the methyl-tert butyl ether (MTBE) method described by Matyash *et al.*[20], with all steps performed in the Captair Pyramid glove box (Erlab, Rowley, MA) under argon gas to prevent exposure to oxygen and subsequent oxidation of lipids. Briefly, 200 μL plasma was added to 80 μL of MS grade H_2_O in a glass tube with a Teflon lined cap, followed by addition of 1.5 mL of molecular grade methanol. The tubes were vortexed, 5 ml of molecular grade MTBE was added, and then the samples were rocked for 1 hr at room temperature. Following the incubation, 1.25 mL of MS grade H_2_O was added and the tubes were vortexed and centrifuged at 1,000 × g for 10 min. The upper organic phase was collected, placed in another glass tube with a Teflon lined cap, and dried under nitrogen gas. Samples were re-suspended in 1 mL of LC-MS grade methanol and topped with high purity argon gas. Samples were stored for no longer than one week at −20°C prior to chromatographic analysis. Samples were put in random order prior to lipid extraction, and again prior to mass spec analysis.

### Liquid chromatography/mass spectroscopy (LC/MS)

Both the targeted and shotgun analysis were performed on lipid extracts, with lipids separated by reversed phase ultra performance liquid chromatography (UPLC) on an Acquity instrument (Waters, Milford MA). Solvent A consisted of an 89:5:5:1 mixture of water, isopropyl alcohol, acetonitrile and 500 mM ammonium acetate respectively. Solvent B consisted of a 50:49:1 mixture of isopropyl alcohol, acetonitrile, and 500 mM ammonium acetate, respectively. All solvents were LC-MS grade (Fisher, Optima LCMS). One microliter injections were loaded to a 1.0 × 100 mm Acquity BEH C8 column held at 50°C with a 140 μL/min flow of 100% solvent A. Solvent A was held at 100% for 0.1 min, followed by a linear gradient to 40% Solvent A over 0.9 min. A second linear gradient to 100% Solvent B was achieved over 10 minutes, followed by a 3 min hold at 100% Solvent B. The chromatographic system was returned to initial conditions via a 0.1 min linear gradient to 100% Solvent A, followed by a 5.9 min equilibration prior to the subsequent injection. Total run time was 20 min. Eluate was introduced to a Q-ToF Micromass spectrometer (Waters/Micromass, Manchester, UK) via electrospray in either the positive or negative ionization mode. Capillary voltages were held at 3000 V and 2200 V in positive and negative ionization modes, respectively. In both modes the sample cone voltage was held at 30 V, the collision energy was held at 7 V, the source temperature was held at 130°C, and the desolvation temperature was held at 300°C. Data was acquired over the 100–1200 m/z range in all analyses, at a rate of 2 scans/sec. The sample set was analyzed in independently randomized technical duplicate for each ionization mode. Both the UPLC and mass spectrometer were operated using MassLynx software version 4.1 (Waters, Milford, MA).

Peak detection and integration were performed using MarkerLynx software (Waters, Milford MA). Chromatographic peaks eluting between 0 and 14 min with m/z values between 100 and 1200 were detected using a mass window of 0.07 Daltons and a retention time window of 0.1 min. Apex Track peak parameter settings were used, with peak width and baseline noise set for automatic calculation. A threshold of 10 counts/scan, a noise elimination level of 6 was implemented to minimize the detection of spectral noise, and the deisotope option was enabled to exclude isotopic peaks from the final data matrix of detected features and their intensities across all samples.

### OPLS modelling

For the shotgun analysis, multivariate statistical analysis was performed using SIMCA-P^+^ software (Umetrics Kinnelton, NJ). We used principal components analysis (PCA), and combined PLS and orthogonal single correction discriminant analysis (OPLS-DA) to analyze the LC/MS data. In short, PCA is an unbiased analysis of the total metabolite content (in our analysis the total plasma lipid profile) that detects variance among samples and provides sample clusters based on similarity of molecular profile. OPLS-DA is an extended form of partial least squares-discriminant analysis (PLS-DA) that, in addition to explaining overall differences between classes, separates predictive and non-predictive variation [21]. Data were modelled to visualize discrimination between the obese and control groups using the OPLS-DA model and scores plot of the first and second components. Goodness of fit was quantified by R^2^X and R^2^Y, and predictability by Q^2^Y.

### Lipid ion analysis

A strict analysis and reduction to specific lipid ions of interest was performed using the models and scores plots from the OPLS-DA analysis, along with the raw LC/MS data. Factors in addition to obesity, including medication use in the control population, could influence and potentially weaken our model because of the heterogeneity of the subject population. However, lipids that emerge as significantly different between non-obese and obese despite the potential weakness of group heterogeneity, best represent the differences due to obesity in the general clinical population. First, a list was compiled of all the ions falling between 0.5 and 1 on the scores plots from both positive and negative mode because these ions were driving the difference between groups in the OPLS-DA model. An additional list was compiled containing all ions that were significantly different between groups based on t-tests comparing feature abundance between groups. All ions on the *t*-test list with a difference of less than 2 fold between obese and control groups were excluded, as were the features with abundances too low to be distinguished as an actual lipid ion, allowing us to narrow the list to approximately the most significantly different 10% of the ions. The remaining ion features on the *t*-test list were cross-referenced with the ion features on the scores plot list. A final list was compiled of ion features that were present on the scores plot list and final *t*-test list. These were the lipid ions that were both driving the difference between groups observed in the OPLS modelling, and that were statistically significantly different between groups. Ions on this list were further examined using tandem mass spectroscopy (MS/MS).

### Tandem mass spectroscopy and lipid identification

Samples were analyzed by MS/MS as described above for the initial LC/MS analysis with the exception of selective mass filtering with the quadrupole for our ions of interest, and a collision energy of 40 V. Fragmentation patterns were analyzed and cross referenced to Lipid Maps mass spectrometry peak prediction resources [22], and matched with published spectra where available for identification of lipids of interest. Standards were commercially available (Avanti Polar Lipids, Alabaster, AL) for three of the tentatively identified lipids of interest, 1-0-1’-(Z)-Octadecenyl-2-Arachidonoyl-sn-Glycero-3-Phosphoethanolamine, 1-Palmitoyl-2-Linoleoyl-sn-Glycero-3-Phosphocholine, and 1-octadecanoyl-2-(5Z, 8Z, 11Z, 14Z-eicosatetraenoyl)-sn-Glycero-3-Phospho-(1’-myo-inositol))and we performed MS/MS on those available to confirm our tentative identifications. Following confirmation of our tentative identifications, we used the ether-linked phosphoethanolamine standard in cell culture treatments to determine if it induced pathological phenotypic changes in endothelial cells.

### Cell culture and lipid treatments

Primary human coronary artery endothelial cells (HCAEC) (Lonza, Walkersville, MD) were grown in endothelial cell growth medium (EBM-2) containing 5% FBS and manufacturer recommended supplemental growth factors, antibiotics, and antimycotics. All assays were performed on cells at 80-100% confluence, between passages 3 and 9. Lipid treatments included normal medium, vehicle controls, and the 1-0-1’-(Z)-Octadecenyl-2-Arachidonoyl-sn-Glycero-3-Phosphoethanolamine (PE) standard. This PE standard was used for endothelial cell treatments as a representative ether-linked lipid because it was the ether-linked standard on our list that was commercially available. Treatment concentration for the PE standard was 25 μg/mL, with all treatments performed for 4 hrs and repeated a minimum of three times in duplicate or triplicate. HCAEC were also treated with L-α-Phosphatidylcholine (PC) standard (Avanti Polar Lipids, Alabaster, AL), and PC standard that was oxidized by exposure to room air for 48 hrs as previously described [13], to confirm the effects of previously described oxidized PAPC in our model.

### Western blot analyses

HCAEC were seeded in 65 mm polystyrene cell culture dishes and grown to at least 80% confluence prior to lipid treatment. Following treatment, cells were scraped in RIPA buffer (50 mM Tris, 0.15 M NaCl, 1% Na deoxycholic acid, 1 mM EGTA, 1% NP40) containing protease and phosphatase inhibitors, and sonicated 3 × 10 sec. Protein concentrations were determined using a BCA assay, and samples were diluted with the appropriate volume of Laemmli sample buffer for loading 25 μg protein per well. Samples were separated on 7.5% polyacrylamide gels at 125 V, and transferred to nitrocellulose membranes (BioRad, Hercules, CA) for 1 hr at 50 V. Membranes were blocked for 1 hr in Superblock (Thermo Scientific, Rockford, IL) and then incubated with primary antibodies against VCAM (1:200) and ICAM (1:200) followed by the appropriate HRP-conjugated secondary antibodies. Membranes were developed by chemiluminescence using SuperSignal West Dura substrate (Thermo Scientific, Rockford, IL), with digital images obtained using the Biospectrum UVP system (Upland, CA). All signals were normalized to β-actin obtained from the same blot, and expressed as the percent of the normal medium control condition.

### ELISA analysis

Monocyte chemoattractant protein-1 (MCP-1) in the cell culture medium was determined using a sandwich ELISA (R & D, Minneapolis, MN). The analysis was performed according to manufacturer instructions. The lower limit of detection was 31.2 pg/mL and average CV was 2.2%.

### Statistical analysis

Modelling and analysis of LC/MS data are described above. Comparison of ion abundance between obese and control groups, and lipid treatment effects on HCAEC were performed using unpaired t-tests. Significance was set *a priori* at p ≤ 0.05.

## Results

### Subject characteristics

There was no difference in average age between groups, but average BMI was significantly higher (p < 0.01) in the morbidly obese group (49.87 ± 11.27 kg/m^2^) compared to control (25.76 ± 4.39 kg/m^2^) and HbA1C was significantly higher (p < 0.01) in the morbidly obese group (6.41 ± 0.35% of total) compared to control (5.15 ± 0.10% of total) (Table 1).

### Global lipidomic analysis

**Figure 1:**
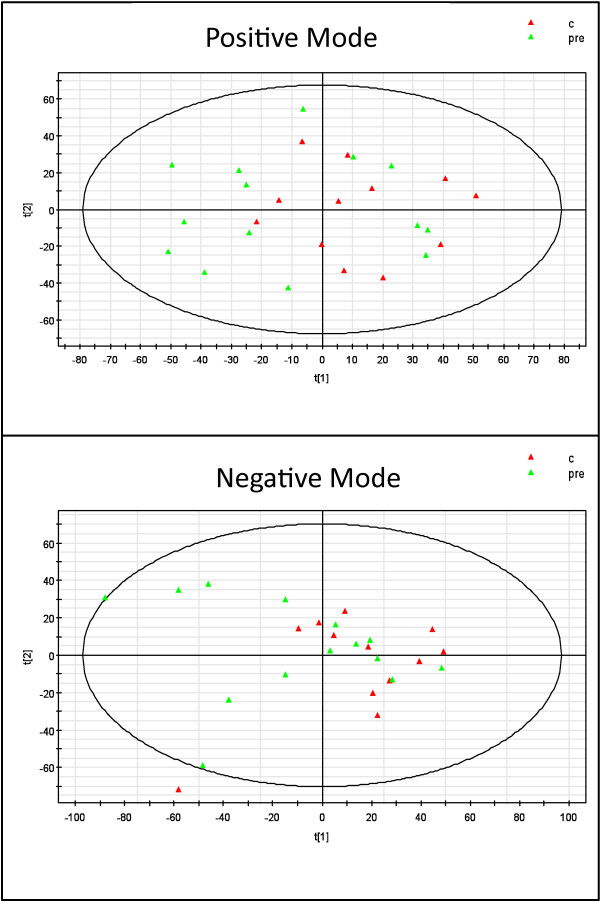
Principle components analysis models for all ions for all subjects detected in positive and negative mode.

**Figure 2:**
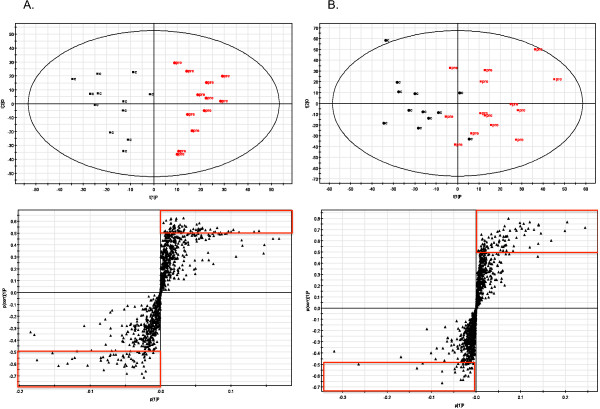
**OPLS-DA models and representative scores plots from positive mode analysis (A) and negative mode analysis (B).** Black markers in the OPLS model represent lean controls, red markers represent obese pre-operative subjects. Each triangle on the scores plot represents an ion that was detected. The red boxes show the ions that were screened for further analysis.

For both the shotgun and targeted analysis we analysed lipid extracts from control and obese subjects using LC/MS. We then began the analysis for the shotgun approach with the principle component analysis (PCA). PCA models (Figure 1) for positive and negative mode poorly classified the control and obese groups, which was not unexpected given the heterogeneity of the groups. The PCA model eliminated outliers prior to the biased OPLS-DA analysis. OPLS-DA modelling was performed on the remaining control and obese lipid profile data obtained in both positive and negative mode. The positive mode model and a representative scores plot are shown in Figure 2A, the negative mode model and a representative scores plot are shown in Figure 2B. R^2^X, R^2^Y, and Q^2^Y of the positive mode model were 0.298, 0.839, and 0.259 respectively, while R^2^X, R^2^Y, and Q^2^Y of the negative mode model were 0.49, 0.658, and 0.216 respectively. Our values are similar to analyses in other heterogeneous diseased human populations [23]. Based on the OPLS-DA models and statistical analysis as described above, a list of 43 lipid ions of interest that were significantly different between groups and driving the group separation in the OPLS-DA model was compiled for further examination (Additional file 1: Table S1), 26 of those were greater in obese subjects.

### Lipid ion of interest identification

MS/MS analysis was performed on all lipid ions of interest. Following MS/MS analysis, screening of spectra, comparison of fragments in Lipid Maps, comparison to published spectra, and elimination of ions that were unidentifiable or became negligible, 22 ions of interest were tentatively identified. Numerous tentatively identified ions were present as multiple adducts, Table 2 shows the final list of ions of interest that were greater in obese subjects and of interest for further study. We discovered that multiple ether-linked phospholipids were significantly higher in the obese group compared to control and were driving group separation in the OPLS-DA model.

**Table 2:** Identifications of ions from MS/MS analysis

**Ion of interest from original LC/MS analysis**	**Tentative ID based on MS/MS analysis**	**P-value**	**Ave (c)**	**Ave (pre)**
750.5399	PE(P-18:0/20:4(5Z,8Z,11Z,14Z))	0.016	8.25	13.73
838.5599	PE(18:2(9Z,12Z)/20:2(11Z,14Z))	0.039	17.67	27.82
829.57	PC(16:0/18:2(9Z,12Z))	0.022	0.01	0.18
802.5599	PC(O-16:0/18:2(9Z,12Z))	0.019	49.22	64.29
816.57	PC(O-16:0/18:2(9Z,12Z))	0.031	161.81	208.10
830.59	PC(O-18:0/18:2(9Z,12Z))	0.003	32.48	45.40
844.5999	PC(O-18:0/18:2(9Z,12Z))	0.005	94.68	130.23
832.5999	PC(O-18:0/18:1(9Z))	0.005	8.85	14.46
868.5999	PC(O-18:0/20:4(5Z,8Z,11Z,14Z))	0.010	41.54	63.87
870.6199	PC(O-18:0/20:3(8Z,11Z,14Z))	0.028	30.86	44.71
885.5499	PI(18:0/20:4(5Z,8Z,11Z,14Z))	0.011	45.66	62.11

p-values were calculated using an unpaired *t*-test comparison of group relative abundance means for each ion from the original run. c = control, pre = obese pre-surgical. Ave (c) and (pre) values are average relative abundance in arbitrary units of each ion for each group from the original run.

**Figure 3:**
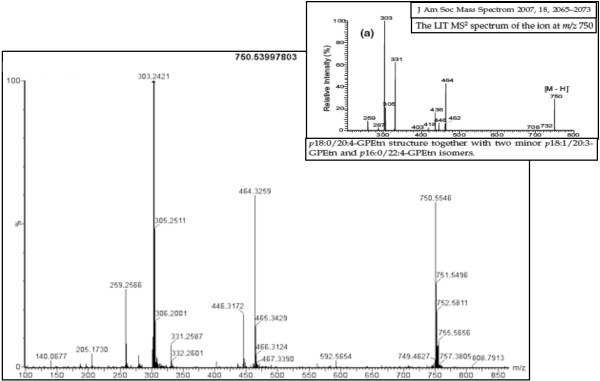
**Representative spectrogram of the ion m/z 750.5399 from MS/MS analysis that was identified as the plasmalogen phosphatidylethanolamine PE(P-18:0/20:4(5Z,8Z,11Z,14Z)), along with a previously published MS/MS spectrogram of the same species.** Peak patterns match with the exception of a fragment of 331 in the previously published spectra resulting from presence of a minor additional isomer.

**Figure 4:**
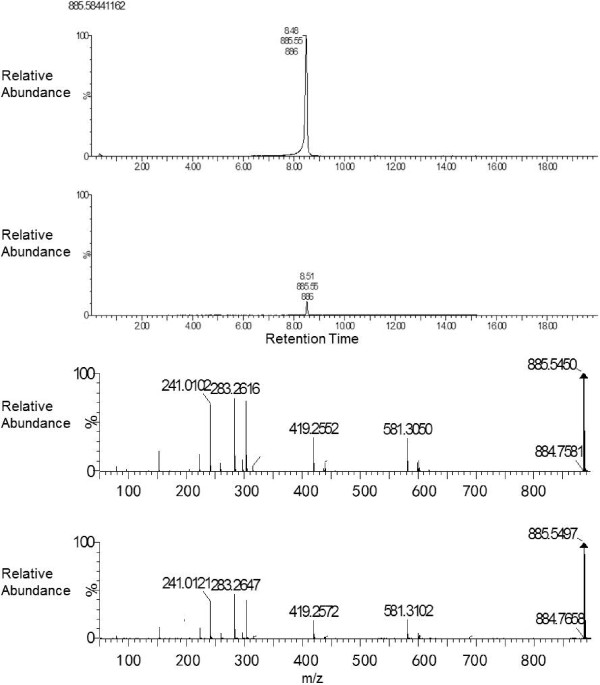
**Chromatography and MS/MS spectra of ion m/z 885.5499 (PI(18:0/20:4(5Z,8Z,11Z,14Z)) from the purchased standard (top chromatogram and spectra) and a sample (bottom chromatogram and spectra).** Fragmentation patterns match at major peaks 885- parent ion, 581- parent ion with loss of sn-2 arachidonic acid side chain m/z 303, 303- liberated arachidonic acid, 283- liberated stearic acid, and 241- inositol head group.

To confirm our tentative ion identifications, where available we compared previously published spectra of our tentatively identified ions to our spectra. Figure 3 shows a representative MS/MS spectra of PE(P-18:0/20:4(5Z,8Z,11Z,14Z)) an ether-linked phosphatidylethanolamine (PE), in one of our samples, that was identified in negative mode, and a previously published spectra of the same ion confirming our tentative identification of that ion. Published spectra for this PE can also be found in Goodenowe *et al.* showing the same fragmentation pattern [24]. We also purchased the standards that were available from our list to analyze via MS/MS and compared the spectra of our unknown ions to the spectra of the standards. Spectra from the standards that were analysed matched the spectra of our unknowns confirming the identity of those ions. Figure 4 shows the chromatography and MS/MS spectra of PI(18:0/20:4(5Z,8Z,11Z,14Z)) from the purchased standard (top chromatogram and spectra) and our sample (bottom chromatogram and spectra). Retention times match, and major identifying peaks include 885 (parent ion) 303 (20–4 arachidonic acid) 283 (18–0 steric acid), and 241 (inositol head group). Because the exceptional finding from the shotgun analysis was the emergence of the group of ether-linked lipids, we next sought to examine what potential effects ether-linked lipids may have on endothelial cells, to obtain insight into the relationship between obesity associated dyslipidemia and vascular disease.

### Endothelial cell response to ether-linked phosphatidylethanolamine and oxidized PAPC

**Figure 5:**
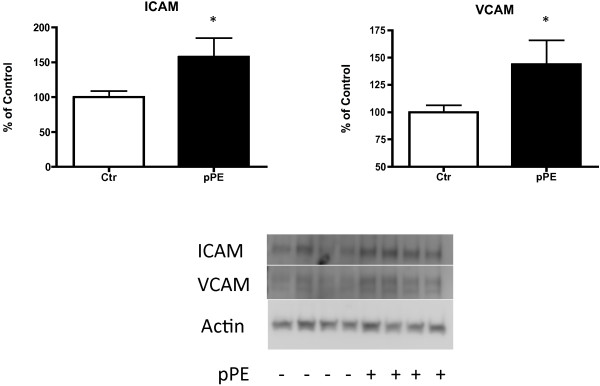
**Effects of lipid treatment on HCAEC cell adhesion molecule protein expression.** pPE increased ICAM protein (158% of control, p = 0.028) and VCAM protein (144% of control, p = 0.038) in HCAEC. Data are presented as Mean ± SE.

**Figure 6:**
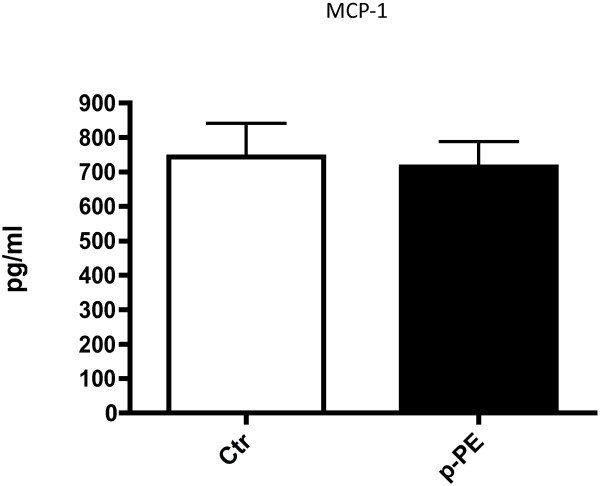
**Effects of lipid treatment on HCAEC MCP-1 secretion.** No differences were detected between treatments. Data are presented as Mean ± SE.

To determine the effect of our identified ether linked lipids on endothelial cells, we used PE-(P-18:0/20:4(5Z,8Z,11Z,14Z)) (p-PE), the only commercially available ether-linked lipid on our final list, to treat HCAEC. p-PE induced increases in the cell adhesions molecules ICAM (158% of control, p = 0.028) and VCAM (144% of control, p = 0.038) (Figure 5). Cell culture medium was collected for analysis of the secreted inflammatory mediator MCP-1. MCP-1 did not differ in medium collected from cells incubated in normal medium, and those that were exposed to p-PE (Figure 6). Because previous studies have described induction of ICAM and VCAM by oxidized phospholipids, we also used oxidized phosphatidylcholine (ox-PC) to treat HCAEC. Ox-PC also induced increases in ICAM (180% of control, p = 0.038) and VCAM (169% of control, p = 0.051), while there was no effect of un-oxidized PC.

### Targeted analysis of oxidized phosphatidylcholine

Because previous *in vitro* data indicate that ox-PC products cause pathological phenotypic changes in endothelial cells, we targeted PC and ox-PC in our LC/MS data from morbidly obese and control plasma using a targeted approach. We scanned all ions in the positive mode analysis for any that could be PC, PGPC, POVPC, or PEIPC based on m/z and abundance. While none of the best potential matches for these lipids were between 0.5 and 1 in our OPLS-DA models, one potential PC match appeared to be significantly higher in obese subjects compared to control (p = 0.040), though the other potential match had a much higher relative abundance (as would be expected for PC because it is the most abundant phospholipid in cells) and was not significantly different. Of the best matches for the ox-PC species, only those for PGPC were close to significantly elevated in the obese compared to control (p = 0.052, and 0.055), while the best potential matches for PEIPC were not significant (p = 0.707, and 0.661) and no ions that could represent POVPC were detected (Table 3).

**Table 3:** Oxidized PAPC products from positive mode analysis

**Phospholipid**	**Expected m/z**	**Best match**	**P-value**	**Ave (c)**	**Ave (pre)**	**Scores plot location**	
PAPC	782.7	11.10_782.6904	0.04	0.029	0.11	x = 0.005	y = 0.4
		8.43_782.5644	0.49	289.14	269.649	x = −0.04	y = −0.1
PGPC	610.2	9.53_610.1888	0.052	0.066	0.262	x = 0.01	y = 0.4
		9.40_610.1828	0.055	15.347	26.288	x = 0.1	y = 0.53
POVPC	594.3	No ions detected at this mass					
PEIPC	828.6	8.31_828.5536	0.707	3.269	3.06	x = 0	y = 0
		8.32_828.6107	0.661	0.191	0.157	x = 0	y = 0

p-values were calculated using an unpaired *t*-test comparison of group means for each ion. c = control, pre = obese pre-surgical. Ave (c) and (pre) values are average relative abundance in arbitrary units of each ion for each group. Scores plot locations represent the coordinates of the location of each ion on the positive mode scores plot shown in Figure 3.

## Discussion

Lipidomics often functions as a hypothesis generating technique where biomarkers of interest emerge from large data sets indicating further analysis of physiological roles of identified features. Because obesity is a major independent risk factor for coronary artery disease, and a major component of that risk is dyslipidemia, we sought to characterize the lipid profiles from morbidly obese human subjects compared to control. We combined *in vivo* and *in vitro* methods to examine the effects of lipids that were identified and elevated in obese subjects on pathophysiology of endothelial cells. The major findings of this study were that a group of ether-linked lipids was elevated in plasma from morbidly obese subjects compared to control, and PE-(P-18:0/20:4(5Z,8Z,11Z,14Z)) increased cell adhesion molecule protein but not the inflammatory mediator MCP-1 in endothelial cells. In addition, PAPC, PGPC, POVPC, and PEIPC were not elevated in our obese group compared to control.

### Ether-linked lipids

There was a significant difference in the lipid profiles between our control and obese subjects. Following our conservative and strict approach to narrowing down the total lipid profile to a small list of lipids of interest, a group of ether-linked phospholipids that were elevated in obese subject plasma compared to control that were driving the separation of groups in our model emerged. Ether-linked lipids are characterized by an ether linkage as opposed to an ester linkage at one or both fatty acid side chains, with the subclass plasmalogens having a vinyl ether linkage. Standard ether-linked lipids as well as plasmalogens both appeared on our list, and the ether-linked phosphatidylethanolamine used in our *in vitro* experiments was a plasmalogen.

Ether-linked lipids have been characterized in macrophages, lens cells, adipose, liver, and serum [25-27]. In the context of metabolic disease, reports detailing ether-linked lipid presence and alteration vary. A recent lipidomic analysis on serum, liver, subcutaneous adipose, and intraabdominal adipose from a group of obese subjects was showed that ether-linked PEs were higher in liver compared to both adipose depots, but ether-linked PCs were higher in adipose depots [26]. In this analysis ester PCs were more abundant in all tissues compared to ether-linked, but ether-linked PEs were more abundant than esters in all tissues. Graesssler *et al.* found a significant increase in multiple PE plasmalogens in individuals with BMI > 27.5, but diminished ether-linked lipids in individuals with increasing HOMA-IR [19]. A comparison of obese and non-obese twins found that more ether-linked phospholipids were diminished in the obese twin compared to the lean than were elevated [28]. Other recent work profiling lipid composition of LDL found that plasmalogen PEs are decreased in metabolic syndrome patients compared to lean, as well as in diabetics compared to lean, and the proportion of plasmalogen PE negatively correlates with waist circumference [29]. Conversely, it has also been reported that plasmalogen presence is greater in hypercholesterolemic subjects [30], and that the ratio of plasmalogen PE to ester PE was increased in a population of diabetic patients without cardiovascular complications compared to controls [31]. In the present study we did not have enough subjects to stratify results based on diabetes in our obese group. Collectively, the work of others as well as the current investigation support further examination of why ether-linked phospholipids vary between lean control and obese subjects and what the role of ether-linked lipids in metabolic disease may be. It may be that the effects and presence of individual ether-linked and plasmalogen PCs and PEs varies depending on individual disease severity and milieu.

### Physiological roles of ether-linked lipids

Ether-linked phospholipids are present in most cell membranes and studies of their function have ranged from signalling in brain cells to antioxidant properties [32-35]. There is a lack of consensus about whether ether-linked lipids are pro-or anti-oxidant and inflammatory. Ether-linked lipids, such as those identified in the current study, are more susceptible to oxidation compared to ester-linked lipids [34,35]. Ether-linked plasmalogens in particular are preferentially oxidized over ester-linked phospholipids, possibly due to structural differences leaving the polyunsaturated sn-2 side chains more exposed [36], and lower bond dissociation energies of ether linkages [33]. Decreased plasmalogens in LDL from patients with the metabolic syndrome and diabetes has been suggested as an indicator of oxidative stress [29]. In the current study it may be that the observed ether-linked lipids are elevated as an antioxidant response to increasing oxidative stress with obesity, and as noted earlier reports vary in regard to increases and decreases in ether-linked lipids based on various symptoms of the metabolic syndrome. Whether ether-linked lipids function as ROS scavengers or are simply more susceptible to oxidation remains to be seen. However if ether-linked lipids are scavengers and are preferentially synthesized in response to a pro-oxidant environment or altered membrane fluidity, this may be of interest in reference to obese and diabetic populations where increased oxidative stress is often a problem.

Ether-linked lipids may be pro-inflammatory and can serve as arachidonic acid reservoirs. Ether-linked lipids, particularly plasmalogens have a high proportion of arachidonic acid at the sn-2 position, and are involved in membrane remodeling as well as intracellular arachidonic acid metabolism [15,16]. Arachidonic acid released from the sn-2 position of ether and ester lipids can be enzymatically oxidized to form prostaglandins, prostacyclin, thromboxanes, and leukotrienes, all of which are pro-inflammatory [37,38]. Ether-linked lipids and oxidation products of ether-linked lipids include proinflammatory lysophospholipids that are platelet activating factor (PAF) precursors, and lysophospholipids independently can be pro-inflammatory and pathological. Lysophosphocholines (LPCs) have been shown to increase plasminogen activator inhibitor-1, a potent prothrombotic and proatherogenic protein [39]. In addition, LPCs can induce endothelial cell expression of cell adhesion molecules, an important step in endothelial cell activation and atherosclerotic progression [40,41].

Ether-linked lipids with arachidonic acid at the sn-2 position serve as PAF synthesis precursors. PAF is an ether-linked species with a choline head group, an ether-linked fatty acid at the sn-1 position and an acetyl group at the sn-2 position that is a potent stimulator of the platelet coagulation cascade, thrombosis, and inflammation. PAF has been implicated in atherogenesis because it activates and recruits inflammatory cells, and in advanced plaque rupture because it initiates the coagulation cascade in platelets (reviewed in [42]). What is unknown is whether PAF formation only occurs in response to external stimuli, or if formation of PAF can be driven by excess substrate availability. The ether-linked phospholipids, including those identified in our analysis with arachidonic acid at the sn-2 position and choline head groups, may serve as substrate for PAF formation and thus be characterized as pro-inflammatory.

### Ether-linked phospholipids and HCAEC

To address whether ether-linked lipids can induce phenotypic changes in endothelial cells, we performed *in vitro* experiments using a commercially available ether-linked PE, PE-(P-18:0/20:4(5Z,8Z,11Z,14Z)), that corresponded to a species we identified as elevated in obese subjects. This PE stimulated cell adhesion molecule expression in human coronary artery endothelial cells a key step in atherogenesis. The increases we observed in cell adhesion molecule expression were similar in magnitude to those observed in endothelial cells following oxidized PAPC treatment [19].

We cannot conclude based on these data that the effects of this plasmalogen phosphatidylethanolamine are representative of other ether-linked lipids, including those ether-linked phosphatidylcholines that were also identified during our analysis. However, our data suggest that this lipid may induce changes in endothelial cells indicative of vascular disease, and support further analysis of the role of ether linked lipids in the vascular endothelium.

### Oxidized phospholipids

The finding that phosphatidylcholine oxidation products that have been shown *in vitro* to induce disease related phenotypic changes in endothelial cells were not elevated in our obese subjects was unexpected. Because oxidized phosphatidylcholine products have been found in modified LDL and atherosclerotic lesions from high fat fed rabbits [6] and oxidized LDL has been found in lesions isolated from human vessels [43], we hypothesized they would be elevated in morbidly obese subjects compared to control. Our findings do not discredit the data indicating detrimental effects of oxidized PAPC on endothelial cells, however oxidized PAPC products did not drive the separation between control and obese groups in our model. Our data support the importance of examining other classes of lipids and presence of oxidized phospholipids in human plasma and LDL.

### Lipidomic and metabolomic analyses

Each lipidomic and metabolomic analysis is performed using unique methods and parameters, and generates unique results contributing to global understanding of group differences and metabolism. Lipidomics has been used recently to compare tissues in subjects with the metabolic syndrome and diabetes, overweight and obese subjects, and gastric bypass patients among others [26,44-46]. While lipidomic and metabolomic studies don’t necessarily address mechanistic questions, information obtained using these techniques provides information on global differences between groups and often drives hypothesis development for mechanistic questions. As such, our analysis revealed a group of ether-linked lipids that are elevated in morbidly obese humans. Further, these findings translated to examination of the pathophysiological roles for this class of lipids, where PE-(P-18:0/20:4(5Z,8Z,11Z,14Z)) induces cell adhesion molecule expression in coronary artery endothelial cells. Limitations in our analysis include a small sample size, and while all preparation and analysis of samples following collection was performed under inert gas, we did not use an internal antioxidant. The complete class of ether-linked lipids found in biological samples is very large and the ether-linked lipids on our list represent a very small percentage, therefore its possible that our data are not generalizable to ether-linked lipids as a class. Regardless, the emergence of the ether-linked lipids of interest is strongly supported. Data in our lipidomic analysis show elevated ether-linked lipids in morbidly obese subjects compared to control. Given the potential pathophysiological roles of ether-linked lipids, our data support further investigation of the role this class may play in obesity associated vascular and metabolic disease.

## Competing interests

The authors have no competing interests to disclose.

## Authors’ contributions

ELD contributed to study design, data collection and analysis, and manuscript preparation. SMP contributed to subject recruitment and data collection. MSH contributed to study design and manuscript preparation and analysis. KLH and BFM contributed to study design, funding, and manuscript preparation and analysis. All authors read and approved the final manuscript.

## Authors’ information

Karyn L Hamilton and Benjamin F Miller Co-principle investigators.

## Supplementary Material

Additional file 1: Table S1 Ions of interest identified in LC/MS analysis that were selected for MS/MS. p-value was calculated using an unpaired *t*-test comparison of group means for each ion. Click here for file
