# Supplementary material for: Lipidomic analysis of human plasma reveals ether-linked lipids that are elevated in morbidly obese humans compared to lean
Source: Diabetol Metab Syndr. 2013 May 14;5:24. doi: 10.1186/1758-5996-5-24 (PMC3663699; doi:10.1186/1758-5996-5-24)
Supplement: Additional file 1: Table S1 — Ions of interest identified in LC/MS analysis that were selected for MS/MS. p-value was calculated using an unpaired t-test comparison of group means for each ion. [file 1758-5996-5-24-S1.pdf]

Supplementary Table 1. Ions of interest identified in LC/MS analysis that were selected for MS/MS. p-value was calculated using an unpaired t-test comparison of group means for each ion. Ave [c] (control) and [pre] (obese pre-surgical) values are average relative abundance of each ion for each group expressed in arbitrary units. C= control, O= obese pre-surgical

**Positive Mode**					**Negative Mode**				
**Ret. Time and Ion**	**P-value**	**Ave [c]**	**Ave [pre]**	**Higher in**	**Ret. Time and Ion**	**P-value**	**Ave [c]**	**Ave [pre]**	**Higher in**
7.99_612.1680	0.046	0.004	0.054	O	9.26_829.5643	0.022	0.0139	0.1783	O
9.52_237.0804	0.033	0.032	0.849	O	9.99_666.6038	0.000	6.9303	11.9490	O
11.51_864.8034	0.012	7.398	11.860	O	9.21_750.5424	0.016	8.2493	13.7307	O
3.35_205.0854	0.025	15.968	12.118	C	11.44_917.7758	0.042	7.4802	13.9198	O
7.19_366.3747	0.018	19.978	12.180	C	9.22_832.6023	0.005	8.8548	14.4645	O
4.52_522.3565	0.041	17.113	12.594	C	9.27_817.6412	0.013	9.6848	15.3987	O
5.20_304.2618	0.044	15.000	12.668	C	9.03_856.6022	0.009	10.4531	16.2154	O
3.35_301.1414	0.044	16.211	13.217	C	11.44_903.7659	0.026	9.9010	16.7782	O
10.09_719.5164	0.013	18.992	13.423	C	7.78_733.5486	0.004	11.9465	17.6002	O
12.12_1128.3085	0.039	6.868	13.549	O	10.31_711.6237	0.030	12.3959	18.1693	O
4.60_930.5785	0.027	21.794	15.768	C	11.25_901.7457	0.038	12.6943	20.4485	O
6.42_338.3426	0.026	25.424	17.522	C	9.34_527.4475	0.036	15.3125	22.4955	O
3.35_149.0242	0.023	25.520	19.548	C	10.30_708.6421	0.021	17.4746	26.3310	O
7.23_344.2969	0.041	28.497	21.618	C	8.11_838.5567	0.039	17.6692	27.8192	O
8.70_734.5697	0.015	27.614	23.764	C	10.30_694.6343	0.013	24.1369	36.2948	O
9.30_811.6669	0.016	43.954	33.723	C	9.02_870.6202	0.028	30.8649	44.7140	O
3.80_702.4395	0.026	63.843	47.216	C	8.91_830.5908	0.003	32.4771	45.3975	O
9.94_705.4997	0.028	65.940	47.692	C	8.01_885.5468	0.011	45.6587	62.1103	O
9.60_813.6826	0.012	81.572	63.719	C	8.89_868.6058	0.010	41.5375	63.8662	O
					8.47_802.5587	0.019	49.2173	64.2907	O
					6.90_493.3676	0.036	121.6714	97.4658	C
					8.91_844.6054	0.005	94.6827	130.2282	O
					8.46_816.5744	0.031	161.8050	208.0984	O
					8.70_599.4461	0.029	729.2035	615.2231	C
